# Temporal correlations in neuronal avalanche occurrence

**DOI:** 10.1038/srep24690

**Published:** 2016-04-20

**Authors:** F. Lombardi, H. J. Herrmann, D. Plenz, L. de Arcangelis

**Affiliations:** 1 Institute of Computational Physics for Engineering Materials, ETH, Zurich, Switzerland; 2 Departamento de Física, Universitade Federal do Ceará, 60451-970 Fortaleza, Ceará, Brazil; 3 Section on Critical Brain Dynamics, NIH, Bethesda, Maryland 20892, USA; 4 Department of Industrial and Information Engineering, Second University of Naples, INFN Gr. Coll. Salerno, Aversa(CE), Italy

## Abstract

Ongoing cortical activity consists of sequences of synchronized bursts, named neuronal avalanches, whose size and duration are power law distributed. These features have been observed in a variety of systems and conditions, at all spatial scales, supporting scale invariance, universality and therefore criticality. However, the mechanisms leading to burst triggering, as well as the relationship between bursts and quiescence, are still unclear. The analysis of temporal correlations constitutes a major step towards a deeper understanding of burst dynamics. Here, we investigate the relation between avalanche sizes and quiet times, as well as between sizes of consecutive avalanches recorded in cortex slice cultures. We show that quiet times depend on the size of preceding avalanches and, at the same time, influence the size of the following one. Moreover we evidence that sizes of consecutive avalanches are correlated. In particular, we show that an avalanche tends to be larger or smaller than the following one for short or long time separation, respectively. Our analysis represents the first attempt to provide a quantitative estimate of correlations between activity and quiescence in the framework of neuronal avalanches and will help to enlighten the mechanisms underlying spontaneous activity.

Bursty dynamics characterizes a wide variety of physical systems. Examples include earthquakes in the earth’s crust^1^, chemical reactions^2^, solar flares^3^,^4^ or Barkhausen noise in ferromagnets^5^, to cite only a few. In neuronal networks, both *in vitro* and *in vivo*, bursts arise from the near synchronous firing of many neurons^6^,^7^,^8^,^9^,^10^,^11^,^12^,^13^,^14^. Burst sequences in ongoing cortical activity generally have an irregular character^15^, similar to physical systems with intermittent dynamics. This translates in a power spectral density (PSD) which exhibits a 1/*f*-like decay, as observed in ongoing neuronal activity measured using the electroencephalogram (EEG), the magnetoencephalogram (MEG) or the local field potential (LFP)^16^,^17^,^18^,^19^.

In the last decade, a particular approach to neuronal synchronization has been proposed, that captures both the intermittent and oscillatory features of spontaneous activity^20^ in the form of ‘neuronal avalanches’^7^,^8^,^11^,^21^,^22^. In neuronal avalanches, activity bursts distribute in sizes and durations according to power laws with specific exponents characteristic of a critical branching process^23^,^24^, thus providing experimental evidences that the brain might self-organize into a critical state^25^. This interpretation has unveiled similarities with other natural phenomena exhibiting scale-free statistics, for instance earthquakes^26^, and suggests a further analysis of the temporal features of neuronal avalanches based on the theory of stochastic processes. In this framework the time intervals between consecutive events, also called inter-event intervals, quiet times^27^,^28^ or waiting times^29^,^30^,^31^, play an important role in the characterization of a process^32^. Here we notice that the definition of inter-event interval or quiet time generally differs from the one of waiting time: While the quiet time is defined as the difference between the ending and starting time of consecutive events, the waiting time describes the time interval between the starting times of consecutive events^27^. These two definitions only coincide when the event durations are negligible compared to quiet times, as it happens for earthquakes. The statistics of quiet times has been recently investigated for neuronal avalanches recorded in cortex slice cultures^28^,^33^. It has been found that the quiet time distribution *P*(Δ*t*) follows a power law at short time scales, namely from a few to 200–300 ms, which indicates that avalanches are temporally correlated if sufficiently close in time. For longer quiet times this distribution is generally characterized by a local maximum that coincides with the characteristic time of underlying slow oscillations, which leads to a peculiar non-monotonic behaviour. The systematic removal of smaller avalanches from experimental time series and the analysis of the resulting quiet time distributions, has further revealed that avalanche occurrence preserves the temporal features of *θ* and *β*/*γ* oscillations and correlations exist between avalanche sizes and quiet times^28^.

The relationship between sizes of synchronized bursts and quiet times is still poorly understood. This problem is related to many open questions about the mechanisms controlling spontaneous activity and its function. In particular, which factors determine the duration or size of a burst and which the duration of quiet times? Several experimental studies suggest that synchronized bursts are terminated by an activity dependent depression of network excitability^12^,^34^,^35^, which is then recovered during quiet times. In this scheme quiet times do not usually correlate with the size of preceding bursts and network excitability is reset to the same value after each burst^13^,^36^,^37^,^38^,^39^. For instance, in the CA3 region of hippocampal slices of adult rats^12^ it has been observed that burst termination is mainly due to the exhaustion of releasable glutamate at recurrent synapses. In this case the burst size is positively correlated with the duration of the previous inter-burst interval, but not with the duration of the following one. This means that the size of a given burst does not influence the duration of the following quiet time. Similar correlations have been measured in several different networks^13^,^37^,^38^,^39^. The positive correlation observed between bursts and preceding quiet times suggests that burst sizes depend on the level of network excitability reached at the end of the quiescence. If this level was determined solely by the length of the quiet time, then it should be reset to the same value after each burst, as the absence of correlations between burst sizes and following quiet times would suggest. In the opposite scenario a larger burst would be followed by a longer quiet time and a positive correlation should be measured between burst sizes and following quiet times. Such a correlation has been only observed in dissociated cultures of rat spinal neurons^40^ and linked to the role of synaptic inhibition in the termination of bursts. More precisely, the length of quiet times has been found to depend on the spike rate during preceding bursts, which suggests a possible role of spike adaptation in the termination of a burst. In summary, excluding a few particular cases, the existing literature suggests that, independently of the intensity and length of a burst, the network excitability decays always to the same resting value. During the following quiescent period excitability is recovered and a new burst is triggered, for example by spontaneous single action potentials or spontaneous miniature synaptic release which can be rapidly amplified and generate larger depolarizing events^6^,^41^.

In order to better understand the relationship between quiescence and activity, here we propose an alternative approach to the study of correlations in spontaneous cortical activity, which has been successfully introduced to address the longstanding problem of magnitude correlations in seismic and solar flare activity^3^,^4^,^42^ and has recently been applied to the analysis of the fMRI BOLD signal^43^,^44^. This approach is based on the method of surrogate data^45^ and consists in a systematic comparison of conditional probabilities evaluated in the real time series with the ones evaluated in a time series where avalanche sizes are randomly reshuffled. Such a procedure is very efficient in case of large statistical fluctuations in the data^3^,^4^,^42^, as for the avalanche time series we analyze. Indeed, in the Supplementary Information (SI) we show that both correlation functions and scatter plots, which are commonly used to investigate the relationship between avalanche sizes and quiet times, are strongly affected by statistical noise, exhibiting fluctuations as large as the ones measured in uncorrelated data sets (Supplementary Figs S1–S3). In particular, scatter plots do not give any clear indication about correlations between avalanche sizes and quiet times (Supplementary Figs S2, S3), as discussed in a previous work^28^.

We consider spontaneous avalanche activity recorded in cortex slice cultures under normal and disinhibited conditions. Firstly we look at correlations between quiet times Δ*t* and avalanche sizes, *s*. We find that avalanche sizes are significantly correlated with both the preceding and the following quiet times. The correlation with the preceding quiet times is stronger at all time scales. Nevertheless, we show that correlations between avalanche sizes and following quiet times are not negligible and are particularly relevant for Δ*t* < 400 ms. In all cases, the specific relationship between quiet times and avalanche sizes is strongly altered in disinhibited cultures. Finally we investigate the relationship between sizes of consecutive avalanches. In normal conditions, we observe that, given an avalanche of a certain size, the following one tends to be smaller if close in time, larger otherwise. In contrast, in disinhibited cultures, an avalanche can be significantly larger than the preceding one, even when avalanches are very close in time.

## Methods

### Experimental setup

All experimental procedures were approved by the Animal Care and User Committee (ACUC) of the National Institute of Mental Health, USA and were carried out in accordance with the approved guidelines. Coronal slices from rat dorsolateral cortex (postnatal day 0–2; 350 μm thick) were attached to a poly-D-lysine coated 60-microelectrode array (MEA; Multichannelsystems, Germany) and grown at 35.5 in normal atmosphere in standard culture medium without antibiotics for 4–6 weeks before recording. Cortex slice cultures retain the cortical architecture of layers and corresponding cell types, as well as the mesoscopic and microcircuit organization in intrinsic cortical connectivity. Three sets of data have been used for the study, recorded in non-driven, driven and disinhibited conditions. The first one has been taken from Beggs *et al*.^8^ and consists of avalanche activity measured from cortex-striatum-substantia nigra triple cultures or single cortex cultures. In short, spontaneous avalanche activity is recorded outside the incubator in standard artificial cerebrospinal fluid (ACSF; laminar flow of 1 ml/min) under stationary conditions for up to 10 hrs. The second set has been taken from Stewart *et al*.^46^. Spontaneous avalanche activity is recorded inside an incubator in 600 μl of culture medium under sterile conditions for at least 5 hrs. The head stage with the MEA is rocked between ±75° with a sinusoidal trajectory and a pause of 10 s at the steepest angles before reversing direction (cycle time 200 s). The exposure to atmosphere at the steepest trajectory angles triggers transient neuronal activity increases. For the third data set, which has been described in Shew *et al*.^47^, bath application of the *GABA*_*A*_-receptor antagonist picrotoxin (PTX; ~3 μmol) is used. In all the three conditions we only consider spontaneous, non-stimulated activity. The spontaneous local field potential (LFP) is sampled continuously at 1 kHz at each electrode and low-pass filtered at 50 Hz. Negative deflections in the LFP (nLFP) are detected by crossing a noise threshold of −3 SD followed by negative peak detection within 20 ms. nLFP times and nLFP amplitudes are extracted. Neuronal avalanches are defined as spatio-temporal clusters of nLFPs on the MEA^8^. A neuronal avalanche consists of a consecutive series of time bins of width 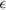 that contain at least one nLFP on any of the electrodes. Each avalanche is preceded and ended by at least one time bin with no activity. Without loss of generality, the present analysis is done with width 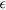 individually estimated for each culture as the average inter nLFP interval on the array at which the power law in avalanche sizes *s, P*(*s*) ~ *s*^−*α*^, yields *α* = 3/2. 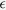 ranged between 3–6 ms for all cultures. Avalanche size is defined as the sum of absolute nLFP amplitudes (μV) on active electrodes or simply the number of active electrodes. We only consider avalanches whose size *s* in μV is larger than a certain threshold *s*_*th*_, i.e. only those avalanches that fall within the power law regime of the size distribution. Avalanche size is then measured in unit of *s*_*th*_.

A quiet time Δ*t* is defined as the time interval between the ending time of an avalanche 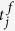 and the starting time 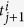 of the following one, namely 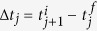.

### Conditional probability approach

The conditional probability approach to correlation analysis is based on the method of surrogate data^45^. Its formal application requires two ingredients, a null hypothesis against which observations are tested, and a discriminating statistic. The null hypothesis is a potential explanation that we seek to show is inadequate for explaining the data. We consider two null hypothesis: (1) quiet times and avalanche sizes are uncorrelated, (2) sizes of consecutive avalanches are uncorrelated. The discriminating statistic here is a conditional probability. If this is significantly different for the observed data than the value expected under the null hypotheses, then the null hypothesis can be rejected. To test this hypothesis we generate surrogate data sets by reshuffling avalanche sizes while keeping the occurrence times fixed.

#### *Correlations between the avalanche size s*_*i*_ *and the following quiet time* Δ*t*_*i*_

**Figure 1:**
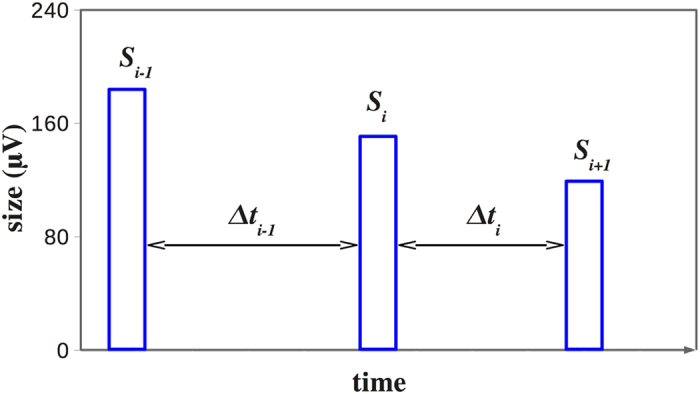
Schematic representation of the avalanche process. A generic avalanche of size *s*_*i*_ is preceded by a quiet time Δ*t*_*i*−1_ and followed by a quiet time Δ*t*_*i*_. Similarly the quiet time Δ*t*_*i*_ separates consecutive avalanches of size *s*_*i*_ and *s*_*i*+1_.

Consider a temporal sequence of avalanches (Fig. 1). Each avalanche *i* has a size *s*_*i*_ and two consecutive avalanches, *i* and *i* + 1, are separated by a quiet time Δ*t*_*i*_. Given two thresholds in size and time, *s*_0_ and *t*_0_, we define the following conditional probability


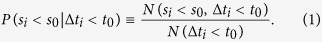


Here *s*_*i*_ is the size of the avalanche preceding Δ*t*_*i*_, *N*(*s*_*i*_ < *s*_0_, Δ*t*_*i*_ < *t*_0_) is the number of avalanches with size *s*_*i*_ < *s*_0_ preceding a quiet time Δ*t*_*i*_ < *t*_0_ and *N*(Δ*t*_*i*_ < *t*_0_) is the number of quiet times Δ*t*_*i*_ < *t*_0_.

**Figure 2:**
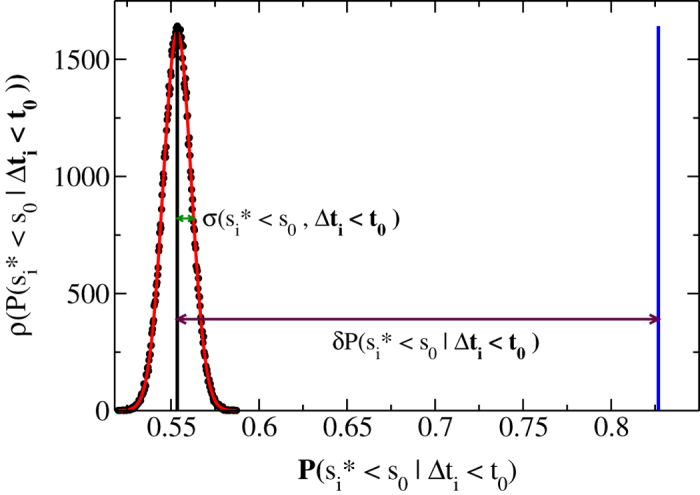
Conditional probability in experimental and reshuffled time series. 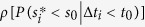
 (black circles) is the distribution of 
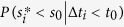
 for *s*_0_ = 300 μV and *t*_0_ = 300 ms, evaluated on 10^5^ realizations of the reshuffled avalanche series. The distribution 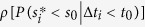 is well fitted by a Gaussian (red curve) with mean value *Q*(*s*_*i*_ < *s*_0_, Δ*t*_*i*_ < *t*_0_) = 0.5536 (black vertical line) and standard deviation *σ*(*s*_*i*_ < *s*_0_, Δ*t*_*i*_ < *t*_0_) = 0.0094. The evaluation of *P*(*s*_*i*_ < *s*_0_|Δ*t*_*i*_ < *t*_0_) in the real avalanche series for the same *s*_0_ and *t*_0_ provides the value 0.8269 (blue vertical line). It follows 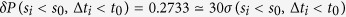, indicating that significant correlations exist between the avalanche size and the following quiet time.

We calculate the conditional probability (1) both for the avalanche time series and for several independent realizations of time series obtained by reshuffling avalanche sizes while keeping fixed their starting and ending times. We denote with 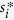 a value of *s*_*i*_ randomly chosen in the entire time series and with 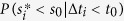 the conditional probability (1) calculated in the reshuffled time series. We evaluate the distribution 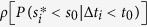 obtained from 10^5^ realizations of reshuffled time series, which, as expected, is Gaussian because 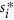 is uncorrelated to Δ*t*_*i*_ by construction. We denote its mean value as 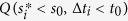 and its standard deviation as 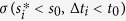 (Fig. 2).

We then compare the mean value 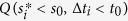 with the corresponding experimental conditional probability *P*(*s*_*i*_ < *s*_0_ | Δ*t*_*i*_ < *t*_0_) defining the difference


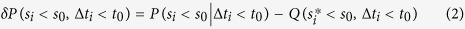


and consider the ratio 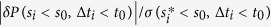 as a measure of significance. If 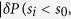 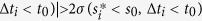 we conclude that, at ~0.05 significance level, significant non-zero correlations exist between *s*_*i*_ and Δ*t*_*i*_ and distinguish two cases: *δP*(*s*_*i*_ < *s*_0_, Δ*t*_*i*_ < *t*_0_) > 0 and *δP*(*s*_*i*_ < *s*_0_, Δ*t*_*i*_ < *t*_0_) < 0. In the first case the number of couples *N*(*s*_*i*_ < *s*_0_, Δ*t*_*i*_ < *t*_0_) satisfying both conditions is significantly larger in the real avalanche series than in the reshuffled one, namely it is more likely to find couples satisfying both conditions in the real rather than in the reshuffled time series. We say that *s*_*i*_ and Δ*t*_*i*_ are positively correlated. Conversely, in the second case the number of couples *N*(*s*_*i*_ < *s*_0_, Δ*t*_*i*_ < *t*_0_) satisfying both conditions is significantly larger in the reshuffled avalanche series than in the real one, namely it is more likely to find couples satisfying both conditions in an uncorrelated rather than in the real time series. In this case we say that *s*_*i*_ and Δ*t*_*i*_ are anti-correlated.

As an example, in Fig. 2 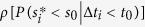 is compared with *P*(*s*_*i*_ < *s*_0_ | Δ*t*_*i*_ < *t*_0_) for a given value of *s*_0_ and *t*_0_.

Finally we introduce the reverse conditional probability


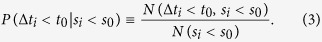


The following relationship between Eqs 1 and 3 holds:


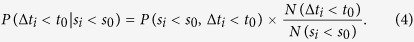


From Eq. 4 it follows that


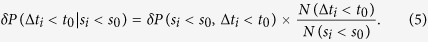


#### *Correlations between the avalanche size s*_*i*+*1*_ *and the preceding quiet time* Δ*t*_*i*_

To study correlations between avalanche sizes *s*_*i*+1_ and the preceding quiet times Δ*t*_*i*_ we consider the following conditional probability


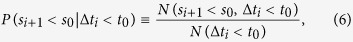


where *s*_*i*+1_ is the size of the avalanche following Δ*t*_*i*_, *N*(*s*_*i*+1_ < *s*_0_, Δ*t*_*i*_ < *t*_0_) is the number of avalanches with size *s*_*i*+1_ < *s*_0_ following a quiet time Δ*t*_*i*_ < *t*_0_ and *N*(Δ*t*_*i*_ < *t*_0_) is the number of quiet times Δ*t*_*i*_ < *t*_0_. The relevant quantity in this case is


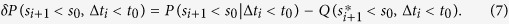


#### Correlations between sizes of consecutive avalanches

For the analysis of correlations between sizes of consecutive avalanches we consider the ratio *s*_*i*+1_/*s*_*i*_ and define the following conditional probability


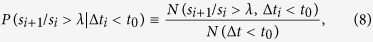


where *λ* is a real number and *N*(*s*_*i*+1_/*s*_*i*_ > *λ*, Δ*t*_*i*_ < *t*_0_) is the number of avalanche couples separated by a Δ*t* < *t*_0_ for which the second avalanche has a size *s*_*i*+1_ larger than *λ* times *s*_*i*_, the size of the first one. Accordingly, in this case the relevant quantity is


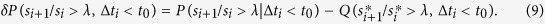


## Results

In the following we analyze avalanche time series recorded in cortex slice cultures under non-driven, driven and disinhibited (PTX) conditions^47^. For each condition results are averaged over all samples. Results for individual cultures are shown in the SI (Supplementary Figs S4–S12). We first study the correlations between quiet times Δ*t* and avalanche sizes, *s*. Then we investigate the relationship between sizes of consecutive avalanches. All results are significant at the 0.05 level (see Methods).

### Correlations between the size *s*_*i*_ and the following quiet time Δ*t*_*i*_

**Figure 3:**
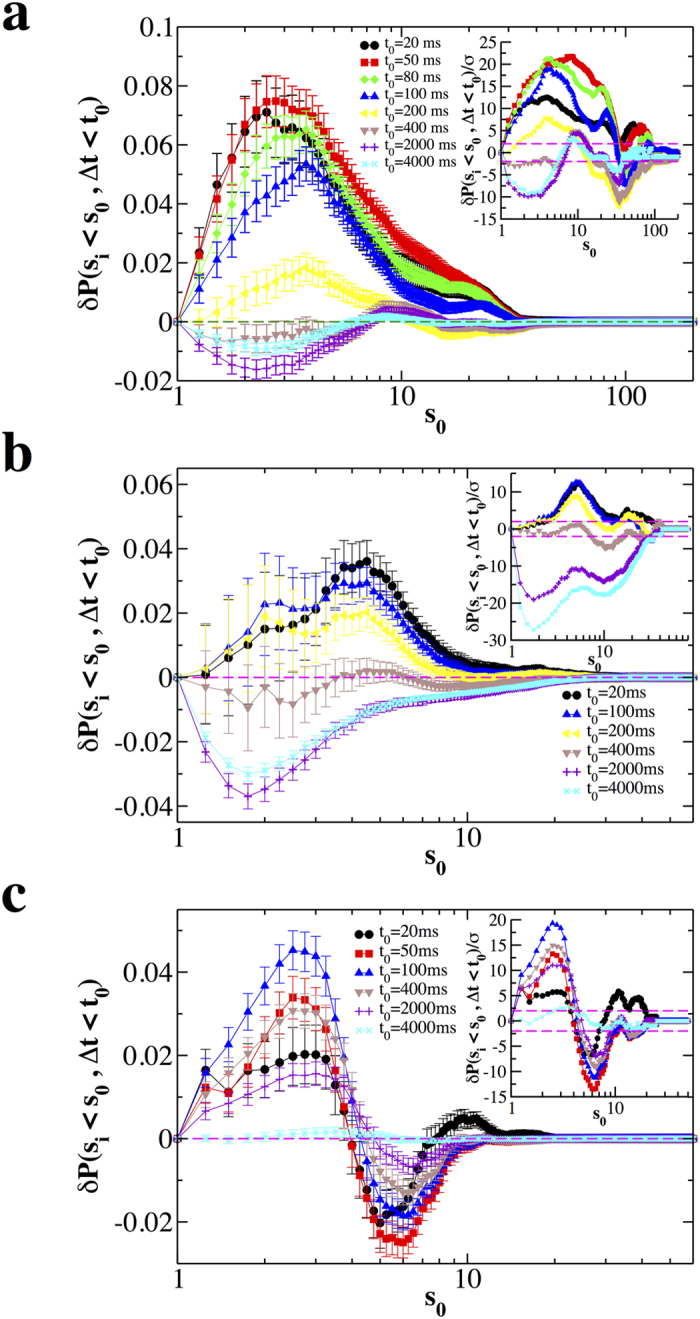
Significant correlations exist between the size *s*_*i*_ and the following quiet time Δ*t*_*i*_. The quantity *δP*(*s*_*i*_ < *s*_0_, Δ*t*_*i*_ < *t*_0_) as a function of *s*_0_ for different values of *t*_0_ and different conditions, non-driven, driven and disinhibited (PTX). The bar on each data point is 2*σ*(*s*_*i*_ < *s*_0_, Δ*t*_*i*_ < *t*_0_). Each curve represents an average over all experimental samples in a given condition. (**a**) Normal; (**b**) Driven; (**c**) Disinhibited. Insets: The ratio *δP*(*s*_*i*_ < *s*_0_, Δ*t*_*i*_ < *t*_0_)/*σ* as a function of *s*_0_ for different values of *t*_0_; dashed lines delimit the interval (−2, 2). In most cases *δP*(*s*_*i*_ < *s*_0_, Δ*t*_*i*_ < *t*_0_)/*σ* is much larger than 2. Therefore these results are significant at a level generally lower than 0.05 and give solid evidences of correlations between avalanches and following quiet times.

To investigate how the quiet time Δ*t*_*i*_ depends on the size *s*_*i*_ of the previous avalanche, we evaluate the quantity *δP*(*s*_*i*_ < *s*_0_ | Δ*t*_*i*_ < *t*_0_) for different values of *s*_0_ and *t*_0_ (see Methods). In Fig. 3 we show *δP*(*s*_*i*_ < *s*_0_ | Δ*t*_*i*_ < *t*_0_) as a function of *s*_0_ for different values of *t*_0_. Both in the non-driven (Fig. 3a) and driven condition (Fig. 3b), for Δ*t*_*i*_ < 200 ms, the quantity *δP*(*s*_*i*_ < *s*_0_, Δ*t*_*i*_ < *t*_0_) takes always positive values beyond error bars, which implies that the probability of finding Δ*t*_*i*_ < 200 ms after an avalanche of size *s*_*i*_ < 30*s*_0_ is larger in the real than in the reshuffled time series. Moreover, since *δP*(*s*_*i*_ < *s*_0_, Δ*t*_*i*_ < *t*_0_) generally decreases by increasing *t*_0_, this probability gradually decreases if one considers avalanches separated by longer Δ*t*.

More specifically, *δP*(*s*_*i*_ < *s*_0_, Δ*t*_*i*_ < *t*_0_) monotonically decreases for *t*_0_ ≥ 50. In the non-driven case, the maximum of *δP*(*s*_*i*_ < *s*_0_, Δ*t*_*i*_ < *t*_0_) is located at 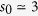 and moves towards larger *s*_0_ with increasing *t*_0_, namely including longer Δ*t* in the analysis. This indicates that larger avalanches tend to be followed by longer quiet times. Indeed, we also notice that, for *t*_0_ > 400, *δP*(*s*_*i*_ < *s*_0_, Δ*t*_*i*_ < *t*_0_) takes always negative values (Fig. 3a,b). This effect is even stronger in the driven case, implying that it is very unlikely to observe small avalanches followed by long quiet times.

In contrast to normal conditions, disinhibited cultures show a consistent crossover from positive to negative values of *δP*(*s*_*i*_ < *s*_0_, Δ*t*_*i*_ < *t*_0_) (Fig. 3c): For all *t*_0_ this quantity is positive for *s*_0_ < 4 and negative otherwise. This implies that positive correlations between avalanche sizes and quiet times exist only for small avalanches, which are significantly correlated not only to short following quiet times as in the normal condition, but also to long following quiet times. In particular, we notice that, for *s*_0_ < 4, *δP*(*s*_*i*_ < *s*_0_, Δ*t*_*i*_ < *t*_0_) first increases with *t*_0_ for *t*_0_ < 100, which indicates an overabundance of small avalanches whose following quiet time is shorter than 100 ms, then slowly decreases and eventually goes to zero for *t*_0_ = 4000 ms (Fig. 3c). In the intermediate range, 4 < *s*_0_ < 10, avalanche sizes are anticorrelated to quiet times, implying that the number of couples satisfying both conditions is smaller than in the uncorrelated time series. Finally all avalanches of size *s* > 10 are uncorrelated with following quiet times and therefore occur randomly in time. This can be understood in terms of the disinhibiting role of the PTX, which alters the occurrence of large avalanches observed under normal conditions.

**Figure 4:**
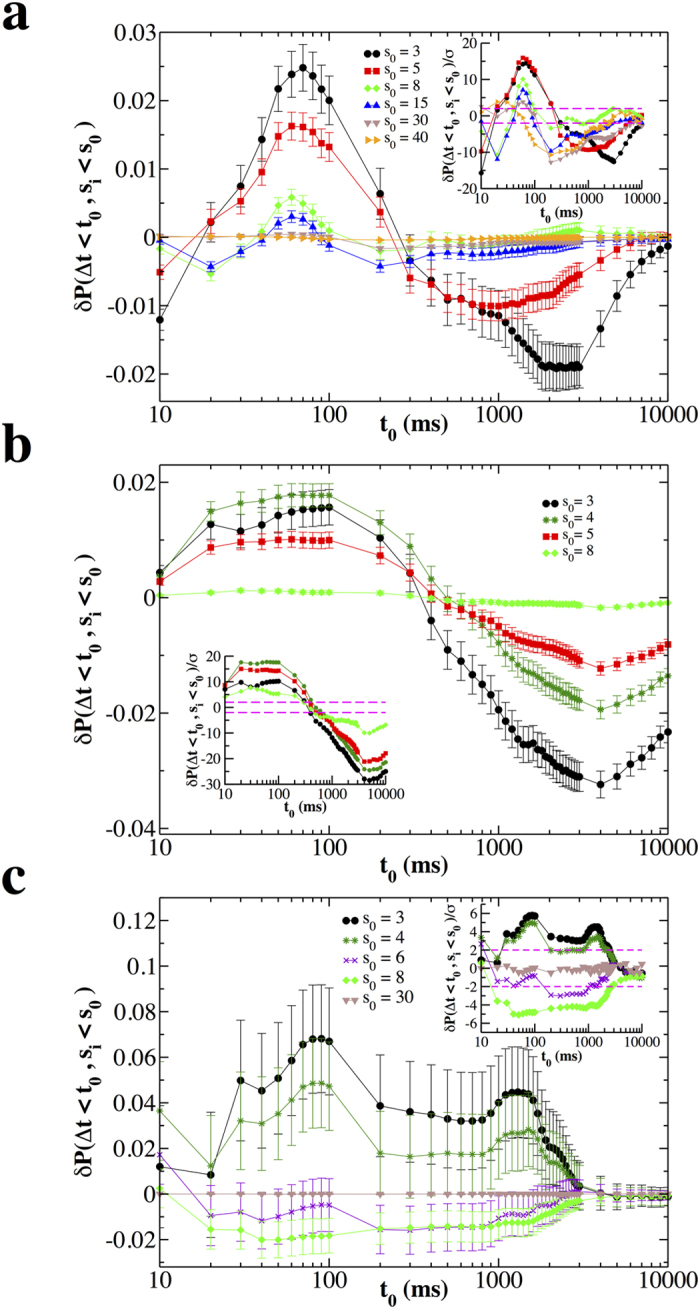
Small avalanches *s*_*i*_ tend to be followed by short quiet times Δ*t*_*i*_ in normal condition. The quantity *δP*(Δ*t*_*i*_ < *t*_0_, *s*_*i*_ < *s*_0_) as a function of *t*_0_ and different values of *s*_0_. The bar on each data point is 2*σ*(Δ*t*_*i*_ < *t*_0_, *s*_*i*_ < *s*_0_). Each curve represents an average over all experimental samples in a given condition. (**a**) Non-driven. (**b**) Driven. (**c**) Disinhibited (PTX). Insets: The ratio *δP*(Δ*t*_*i*_ < *t*_0_, *s*_*i*_ < *s*_0_)/*σ* as a function of *t*_0_ for different values of *s*_0_; dashed lines delimit the interval (−2, 2). In most cases, *δP*(Δ*t*_*i*_ < *t*_0_, *s*_*i*_ < *s*_0_)/*σ* is much larger than 2. Therefore these results are significant at a level generally lower than 0.05 and give solid evidences of correlations between avalanches and following quiet times.

To better enlighten the relationship between *s*_*i*_ and Δ*t*_*i*_ we consider the quantity *δP*(Δ*t*_*i*_ < *t*_0_, *s*_*i*_ < *s*_0_) (Fig. 4). In the normal conditions, for *s*_0_ < 5, *δP*(Δ*t*_*i*_ < *t*_0_, *s*_*i*_ < *s*_0_) is positive for *t*_0_ < 200 ms and negative otherwise, decreasing, in absolute value, for increasing *s*_0_ values (Fig. 4a,b). This behaviour and the relative maximum observed around *t*_0_ = 100 ms, indicate that there is an overabundance of small avalanches whose following quiet time is shorter than 100 ms, suggesting that small avalanches tend to be followed by short, rather than long quiet times. On the other hand, in the disinhibited condition (Fig. 4c), for fixed *s*_0_ values, *δP*(Δ*t*_*i*_ < *t*_0_, *s*_*i*_ < *s*_0_) does not transition from positive to negative values and is either always positive or negative, depending on *s*_0_. This indicates that the correlation sign depends on avalanche size, whereas in the normal condition it depends on quiet times (Fig. 3a,b). For *s*_0_ < 4 *δP*(Δ*t*_*i*_ < *t*_0_, *s*_*i*_ < *s*_0_) is positive and, considering error bars, it is nearly constant over a large range of *t*_0_. Therefore we do not observe a very close relationship between small avalanches and short following Δ*t*. Moreover, since for *s*_0_ > 6 *δP*(Δ*t*_*i*_ < *t*_0_, *s*_*i*_ < *s*_0_) is always negative or zero, Fig. 4c clearly shows that, in the disinhibited condition, large avalanches are generally uncorrelated to following Δ*t*.

### Correlations between the size *s*_*i*+1_ and the preceding quiet time Δ*t*_*i*_

**Figure 5:**
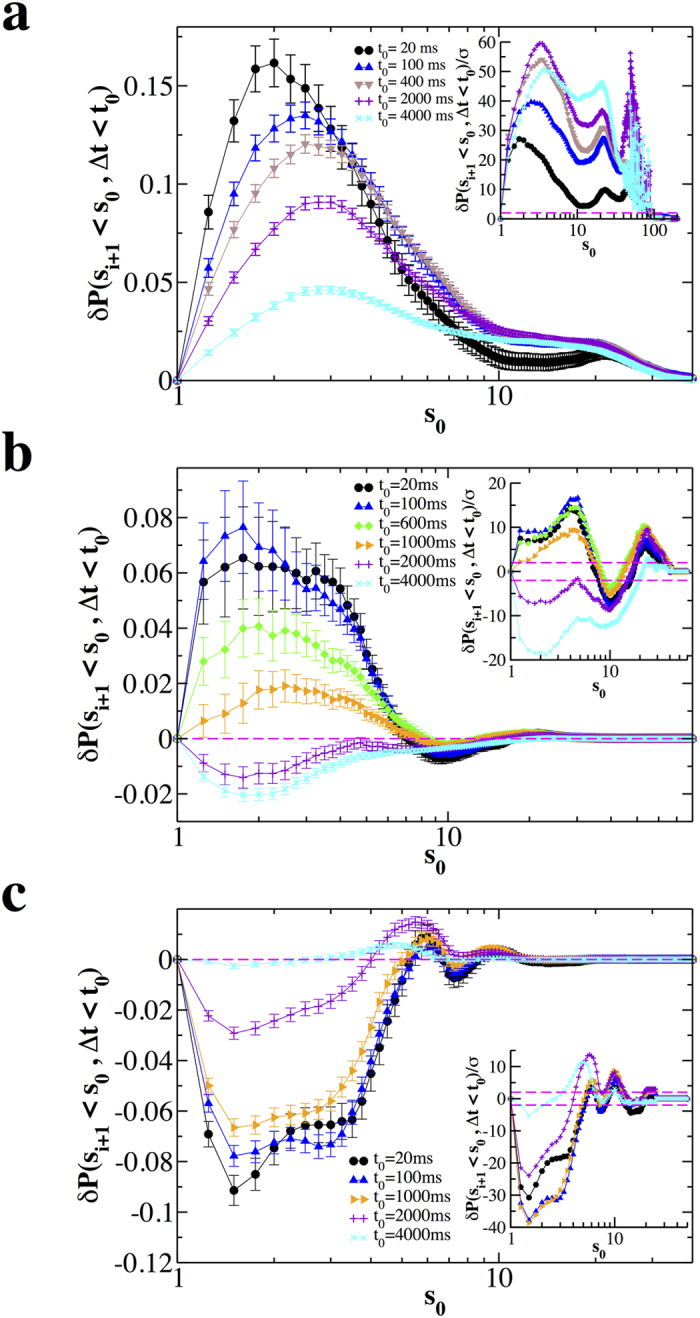
Relation between the quiet time Δ*t*_*i*_ and the size *s*_*i*+1_ of the following avalanche. The quantity *δP*(*s*_*i*+1_ < *s*_0_, Δ*t*_*i*_ < *t*_0_) as a function of *s*_0_ for different *t*_0_ values and different conditions, non-driven, driven and disinhibited (PTX). Each curve represents an average over all experimental samples in a given condition. The bar on each point is 2*σ*(*s*_*i*+1_ < *s*_0_, Δ*t*_*i*_ < *t*_0_). (**a**) Non-driven; (**b**) Driven; (**c**) Disinhibited (PTX). Insets: The ratio *δP*(*s*_*i*+1_ < *s*_0_, Δ*t*_*i*_ < *t*_0_)/*σ* as a function of *s*_0_ for different values of *t*_0_; dashed lines delimit the interval (−2, 2). In most cases *δP*(*s*_*i*+1_ < *s*_0_, Δ*t*_*i*_ < *t*_0_)/*σ* is much larger than 2. Therefore these results are significant at a level generally lower than 0.05 and give solid evidences of correlations between avalanches and preceding quiet times.

Next we study the relation between the quiet time Δ*t*_*i*_ and the size *s*_*i*+1_ of the following avalanche. In this case we analyze the quantity *δP*(*s*_*i*+1_ < *s*_0_, Δ*t*_*i*_ < *t*_0_), which we show in Fig. 5 as a function of *s*_0_ for several values of the threshold *t*_0_. Firstly, we discuss the non-driven condition (Fig. 5a) and notice that *δP*(*s*_*i*+1_ < *s*_0_, Δ*t*_*i*_ < *t*_0_) is always positive and decreases going from *t*_0_ = 20 ms to *t*_0_ = 4000 ms. When avalanches separated by larger time intervals are progressively included in the analysis, the maximum of *δP*(*s*_*i*+1_ < *s*_0_, Δ*t*_*i*_ < *t*_0_) consistently shifts towards larger *s*_0_ values, suggesting that the longer the quiet time the larger the following avalanche.

In the driven condition *δP*(*s*_*i*+1_ < *s*_0_, Δ*t*_*i*_ < *t*_0_) exhibits a similar behaviour (Fig. 5b), namely it decreases for increasing *t*_0_ and eventually becomes negative. On the other hand, an important difference with the non-driven case is that the maximum of *δP*(*s*_*i*+1_ < *s*_0_, Δ*t*_*i*_ < *t*_0_) becomes very wide in the range 1 < *s*_0_ < 4 and then rapidly goes to zero for larger *s*_0_ values. This behaviour results from the average over cultures and does not characterize single experimental samples, for which *δP*(*s*_*i*+1_ < *s*_0_, Δ*t*_*i*_ < *t*_0_) has a well localized maximum (see Supplementary Fig. S8). Therefore, when looking at the single culture, the same conclusions drawn for the normal non-driven condition hold for the driven one. However the position of the maximum of *δP*(*s*_*i*+1_ < *s*_0_, Δ*t*_*i*_ < *t*_0_) varies between *s*_0_ = 2 and *s*_0_ = 4, depending on the sample (see Supplementary Fig. S8). This effect could be accounted for by the larger average rate of LFPs^48^ per electrode due to the slow tilting of cultures, which might lead to large avalanches also after relatively short quiet times.

Differently from the normal condition, in the presence of PTX *δP*(*s*_*i*+1_ < *s*_0_, Δ*t*_*i*_ < *t*_0_) is always negative (Fig. 5c) until avalanche sizes and quiet times become uncorrelated, for Δ*t* ≃ 4 s. This implies that the shorter the quiet time the more unlikely it is to observe small following avalanches. It is worth noticing that, for *t*_0_ < 1000 ms, *δP*(*s*_*i*+1_ < *s*_0_, Δ*t*_*i*_ < *t*_0_) is nearly independent of *t*_0_ and, as a consequence, so is the relationship between Δ*t* and *s*_*i*+1_.

### Correlations between avalanche sizes

**Figure 6:**
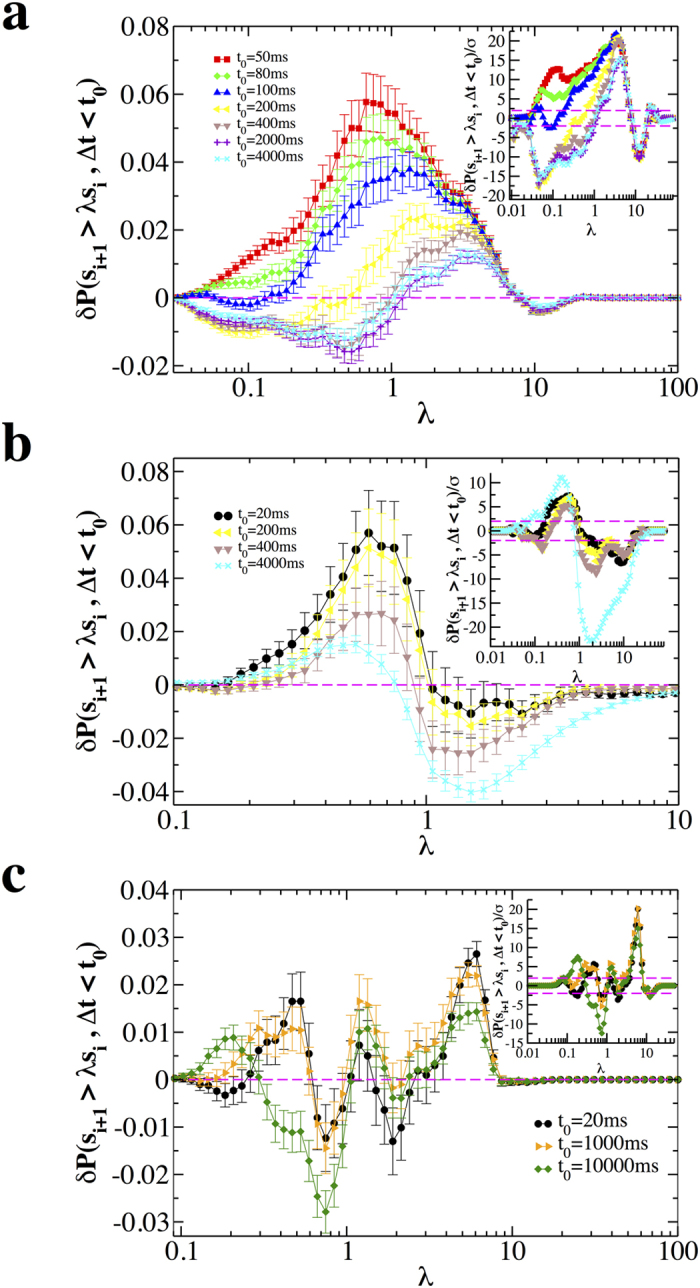
Sizes of consecutive avalanches are correlated. The quantity *δP*(*s*_*i*+1_ > *λs*_*i*_, Δ*t*_*i*_ < *t*_0_) as a function of *λ* for different values of *t*_0_ and different conditions, non-driven, driven and disinhibited (PTX). The bar on each data point is 2*σ*(*s*_*i*+1_ > *λs*_*i*_, Δ*t*_*i*_ < *t*_0_). Each curve represents an average over all experimental samples in a given condition. (**a**) Non-driven; (**b**) Driven; (**c**) Disinhibited (PTX). Insets: The ratio *δP*(*s*_*i*+1_ > *λs*_*i*_, Δ*t*_*i*_ < *t*_0_)/*σ* as a function of *s*_0_ for different values of *t*_0_; dashed lines delimit the interval (−2, 2). In most cases *δP*(*s*_*i*+1_ > *λs*_*i*_, Δ*t*_*i*_ < *t*_0_)/*σ* is much larger than 2. Therefore results are significant at a level generally lower than 0.05 and give solid evidences of correlations between consecutive avalanches.

In previous sections we have shown that the size of an avalanche is significantly correlated with both the previous and the following quiet time. Here we investigate the relationship between successive synchronous firing events, namely between sizes of consecutive neuronal avalanches. In particular, we ask what is the probability of finding an avalanche whose size *s*_*i*+1_ is larger than *λ* times the size *s*_*i*_ of the previous one after a quiet time shorter than *t*_0_ and consider the quantity *δP*(*s*_*i*+1_ > *λs*_*i*_, Δ*t* < *t*_0_). In Fig. 6 we plot this quantity as a function of *λ* for different values of *t*_0_. We first discuss the non-driven case (Fig. 6a). We observe that *δP*(*s*_*i*+1_ > *λs*_*i*_, Δ*t* < *t*_0_), and in particular its maximum, decreases by increasing *t*_0_, meaning that, correlations in size depend on the time interval separating two avalanches. At short time scales, namely for *t*_0_ < 100 ms, *δP*(*s*_*i*+1_ > *λs*_*i*_, Δ*t* < *t*_0_) is always positive or zero: Its maximum is located around 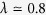, suggesting that, for close-in-time consecutive avalanches, the second one tends to be smaller than the first one. Conversely, for *t*_0_ > 200, *δP*(*s*_*i*+1_ > *λs*_*i*_, Δ*t* < *t*_0_) is negative for *λ* < 1 and reaches its maximum at *λ* > 1. Therefore for larger temporal separation, the second avalanche can be substantially larger than the previous one (Fig. 6a).

In the driven condition (Fig. 6b) *δP*(*s*_*i*+1_ > *λs*_*i*_, Δ*t* < *t*_0_) is always positive for *λ* < 1 and negative for *λ* > 1, implying that, independently of the time separation, it is unlikely to observe an avalanche larger than the previous one. At short time scales, the scenario resembles the one observed in the non-driven case. Indeed, for *t*_0_ < 200, *δP*(*s*_*i*+1_ > *λs*_*i*_, Δ*t* < *t*_0_) is sharply peaked around 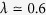, independently of *t*_0_. This implies that correlation features do not depend on time separation for Δ*t* <200 ms. For larger values of *t*_0_, contrary to the non-driven case, *δP*(*s*_*i*+1_ > *λs*_*i*_, Δ*t* < *t*_0_) remains positive for *λ* <1 and therefore, also for large temporal separations, an avalanche tends to be smaller than the preceding one.

In the presence of PTX, the relationship between sizes of consecutive avalanches exhibits a dramatic change (Fig. 6c). Indeed *δP*(*s*_*i*+1_ > *λs*_*i*_, Δ*t* < *t*_0_) repeatedly transitions from positive to negative values and viceversa, showing positive peaks both for *λ* < 1 and *λ* > 1. Therefore the sign of correlations depends on the ratio *λ* between consecutive avalanche sizes and, contrary to the normal condition (Fig. 6a,b), *δP*(*s*_*i*+1_ > *λs*_*i*_, Δ*t* < *t*_0_) is nearly independent of the time separation Δ*t* between consecutive avalanches (Fig. 6c). The behaviour of *δP*(*s*_*i*+1_ > *λs*_*i*_, Δ*t* < *t*_0_) is strongly sample dependent, namely individual cultures may exhibit either single or multiple peaks (see Supplementary Fig. S12). Therefore, the functional behaviour observed in Fig. 6c results from the average over cultures and does not characterize single experimental samples. Importantly, all cultures exhibit a relative maximum for *λ* > 1 and for *t*_0_ values ranging from 20 ms to 4000 ms (Supplementary Fig. S12), evidencing that in the disinhibited condition, the avalanche process is extremely unbalanced and, given two consecutive avalanches, the second one can be much larger than the first avalanche. We want to stress here that, in contrast to normal conditions, this holds also for consecutive avalanches separated by short quiet times and represents a main feature of disinhibited networks. In other words, when inhibitory transmission is reduced by PTX, the length of the quiescent period does not play a significant role in the dynamics.

## Discussion

We have investigated the relationship between quiescence and synchronous bursts in the spontaneous local field potential activity of cortex slice cultures measured with planar integrated microelectrode arrays. Organotypic cortex cultures maintain their main neuronal as well as non-neuronal cell types and cortical layers (refs 6, 26, 46 and 47 and references therein). Specifically, they display spontaneous up- and down-states at low average firing rate similarly to what has been described for the *in vivo* resting state and avalanche statistics that follows those described *in vivo*^7^,^20^. In our avalanche analysis of this *in vitro* system, the existence of a non-zero *δP* indicates that the quiet time Δ*t*_*i*_ depends on the size of the preceding avalanche and, at the same time, determines the size of the following one. This relationship between quiet times and avalanche sizes appears to depend on the condition of the cultures and therefore on the state of the system, which is critical in the normal and supercritical in the disinhibited condition^8^,^47^. In the critical state, small avalanches tend to be followed by short, rather than long quiet times. On the other hand, in the supercritical state small avalanches can be followed by a very wide range of Δ*t*, up to 2 s, while large avalanches are uncorrelated with following quiet times. In addition, in the normal, critical state, longer recovery periods are needed for the system to generate large avalanches, whereas in the disinhibited condition short periods of quiescence can be followed by large avalanches. In sum, suppressing inhibition disrupts the relationship between quiescence and bursts that characterizes cortical networks at criticality. The different correlations in the presence of PTX suggest that inhibition plays an important role in the temporal organization of spontaneous activity in the critical state, that is in avalanche termination and triggering. In other words the relationship between quiescence and synchronous firing observed in the normal condition reflects a specific balance between excitation and inhibition, which characterizes the critical state^33^,^47^.

A related problem, which can help understanding spontaneous cortical dynamics, is the one of correlations between sizes of consecutive avalanches and their dependence on Δ*t*. The first important question is whether avalanche sizes are correlated and whether these correlations depend on Δ*t*. Here we have shown that the size *s*_*i*+1_ of an avalanche following Δ*t*_*i*_ positively correlates with the size *s*_*i*_ of the preceding one and that this correlation depends on Δ*t*, particularly in the normal condition. Indeed curves corresponding to different values of *t*_0_ clearly differ and the larger *t*_0_, the smaller the correlations. More precisely, when the time interval elapsed between two consecutive avalanches is shorter than ≃200 ms, the first avalanche tends to be larger than the following one. Conversely, for longer time separation the first avalanche tends to be smaller than the following one. In the case of network disinhibition, this relationship is strongly altered and an avalanche can be considerably larger than the previous one, independently of the quiet time. The fact that this relationship is altered in the disinhibited condition indicates that its underlying mechanism involves inhibitory neurotransmitters. When the normal, critical balance between excitation and inhibition is altered, the relationship between activity and quiescence changes. For short quiet times, the preceding avalanche tends to be small in both cases. On the other hand, the size of the following avalanche is correlated to the previous Δ*t* in the normal condition and anti-correlated otherwise. This is at the origin of the differences in size correlations we observe for short Δ*t*s.

Recent studies demonstrate that spontaneous activity is linked to stimulus-evoked activity^49^,^50^,^51^,^52^,^53^,^54^,^55^,^56^. For example, the large variability observed in the response to an external stimulus can be accounted for by the fluctuations in the pre-stimulus ongoing activity^50^. In other words, ongoing and evoked activity do interact with each other. An interesting point that would deserve further investigation is how the structure of temporal correlations in spontaneous activity influences the network response, especially the correlations in neural firing induced by external stimuli. Importantly, it has been shown that neuronal networks maximize the range of stimulus intensity they can process^47^,^57^, also called dynamic range, at criticality, namely for the specific temporal correlations we measured in balanced networks. Conversely, when inhibition is suppressed and these correlations change as discussed above, networks fail to discriminate larger inputs^47^. The analysis we performed therefore allows to link the network response to the pre-stimulus temporal correlations in the framework of neuronal avalanches.

The relationship between quiet times and the size of the following synchronized bursts has been previously reported in several experimental studies^13^,^36^,^37^,^38^,^39^. However there was basically no evidence in favor of quiet times being correlated with the size of preceding bursts, except for the work of Streit *et al*.^40^ on dissociated cultures of rat spinal neurons. This has led to a model of burst dynamics where network excitability has a resting value which is independent of the previous network firing: No matter the burst size, the network resets to the same level of excitability. Then, the following quiescent period determines the amount of resources available for the next burst and, as a consequence, its size and duration. This conclusion is mainly based on the analysis of scatter plots or similar quantities which can be strongly affected by statistical fluctuations, intrinsic to experimental data. For instance, in our specific case, the scatter plots do not give any clear indication about correlations between sizes and quiet times^28^.

In order to avoid this problem, our technique compares conditional probabilities evaluated in real and reshuffled time series^42^,^43^. This method allowed us to show that correlations between avalanche sizes and following quiet times can be significant, which suggests that the level of excitability of the network might depend on previous firing activity and opens two possible scenarios: (1) The network excitability has a resting value that is activity-dependent, namely the larger the previous avalanche the lower this value, whereas the recovery mechanism towards the following avalanche does not depend on past activity; (2) After an avalanche, the network excitability decays always to the same value, whereas the recovery mechanism depends on past activity in such a way the larger the previous avalanche the longer the quiescent period preceding the triggering of the following one.

In both cases the recovery period keeps memory of past network firings. This point is further supported by the analysis of size correlations. First, sizes of consecutive avalanches are significantly correlated. How could they possibly correlate if the recovery period did not keep track of past activity? Second, correlations not only quantitatively but also qualitatively change when the time separation between consecutive avalanches increases: While for short time separations an avalanche tends to be smaller, after long quiet times it tends to be larger than the previous one. This clearly indicates that quiet times depend on network activity, as suggested by numerical simulations in Lombardi *et al*.^33^.

Indeed the quiet time distribution can be reproduced by a model where the level of excitability is homeostatically driven by the network activity^33^. In particular, after a large avalanche, the network is hyperpolarized proportionally to its past activity and transitions into a down-state. The synaptic activity during a down-state is modeled as a random process that slowly brings the system back into an up-state. As a result, numerical distributions show the same functional behaviour measured experimentally. This suggests that excitability depends on past activity and that this memory becomes weaker after long recovery time, because of the randomness of spontaneous synaptic vesicle releases^33^.

## Additional Information

**How to cite this article**: Lombardi, F. *et al*. Temporal correlations in neuronal avalanche occurrence. *Sci. Rep.* **6**, 24690; doi: 10.1038/srep24690 (2016).

## Supplementary Material

Supplementary Information
