# Supplementary material for: Temporal correlations in neuronal avalanche occurrence
Source: Sci Rep. 2016 Apr 20;6:24690. doi: 10.1038/srep24690 (PMC4837393; doi:10.1038/srep24690)
Supplement: Supplementary Information [file srep24690-s1.pdf]

# Temporal correlations in neuronal avalanche occurrence: Supplementary Information

F. Lombardi[1,*], H.J. Herrmann[1,2], D. Plenz[3], L. de Arcangelis[4],
1 Institute of Computational Physics for Engineering Materials, ETH, Zurich, Switzerland
2 Departamento de Física,Universitade Federal do Ceará, 60451-970 Fortaleza,Ceará,Brazil
3 Section on Critical Brain Dynamics, NIH, Bethesda, Maryland 20892,USA
4 Department of Industrial and Information Engineering, Second University of Naples, INFN Gr. Coll. Salerno, Aversa(CE),Italy
* E-mail: fabrizio.lombardi@ifb.baug.ethz.ch

## Avalanche size correlation function

The average correlation function $C(\tau)$ is defined as

$$C(\tau) = \frac{1}{T}\sum_{t=0}^{T}(s(t+\tau) - \overline{s(t)})(s(t) - \overline{s(t)})/\sigma^2, \qquad (1)$$

where $\overline{s(t)}$ is the average avalanche size and $T$ is the total recording time.
In order to evaluate the role of statistical noise on avalanche sequences, we calculate the correlation function $C(\tau)$ in experimental and in reshuffled time series (Fig. S1). Both in the non-driven and driven condition, the two correlation functions strongly differ at small $\tau$: While $C(\tau)$ fluctuates around zero for the reshuffled data sets, it is clearly larger than zero for the experimental data sets (Fig. S1). This suggests that correlations between avalanches are relevant on short time scales. On the other hand, for $\tau > 100\,\mathrm{ms}$, $C(\tau)$ exhibits strong fluctuations over a range comparable to the uncorrelated data sets, which makes difficult to extract information about correlations at large time intervals.

As in the normal conditions, in the disinhibited cultures $C(\tau)$ differs from zero and shows peaks localized at $\tau < 100\,\mathrm{ms}$, which can be related to $\theta$, $\beta/\gamma$ oscillations. In *Lombardi et al.* 2014 (Front. Syst. Neurosci. 8:204), we have shown that similar peaks emerge in the quiet time distribution $P(\Delta t)$ when small avalanches are removed from the time series, which constitutes a first indication of correlation between avalanche sizes and quiet times (see *Lombardi et al.* (2014), Front. Syst. Neurosci. 8:204). Finally, for $\tau > 100\,\mathrm{ms}$ we notice that the correlation function shows few more peaks, but in general fluctuations are comparable to the ones of the reshuffled data sets (Fig. S1).

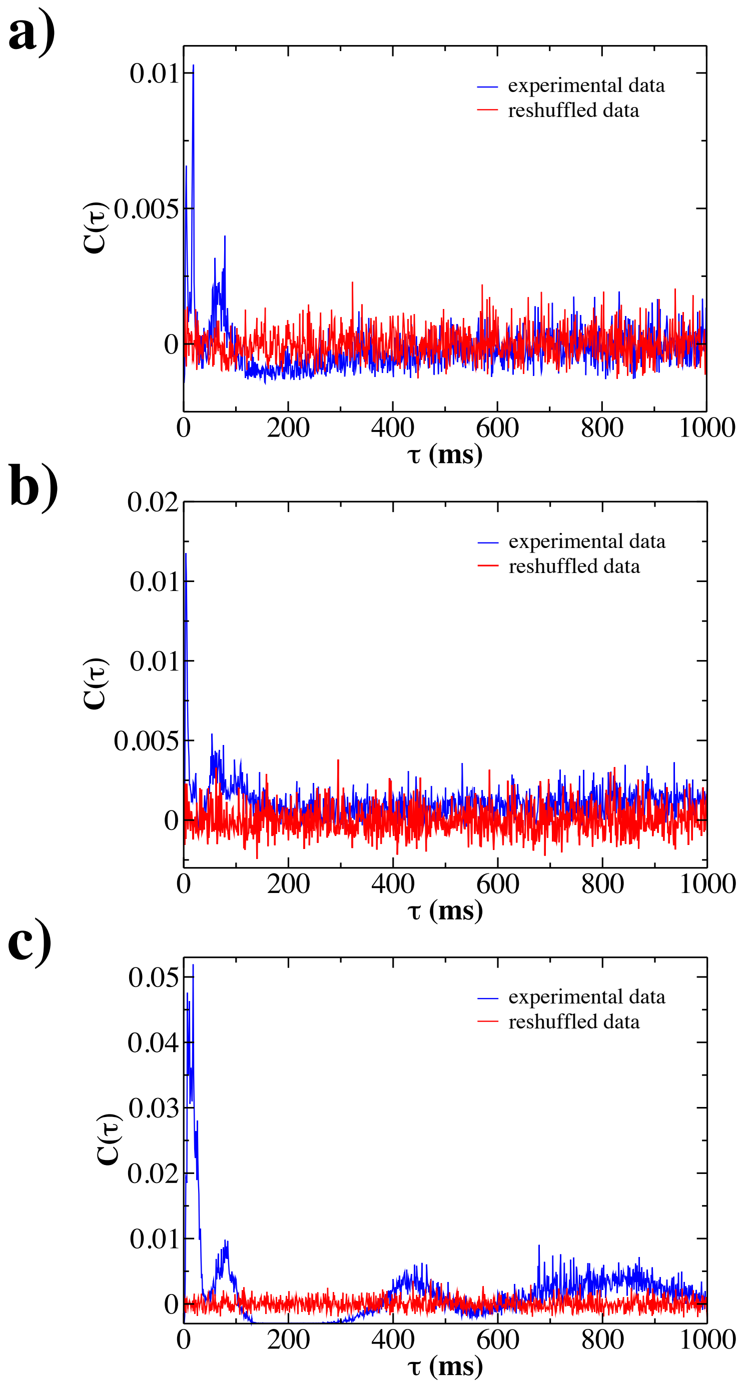


**Supplementary Fig. S1.** Correlation function for avalanche sizes in the experimental and in the reshuffled data set. (a) Non-driven; (b) Driven; (c) Disinhibited (PTX).

# Scatter plots

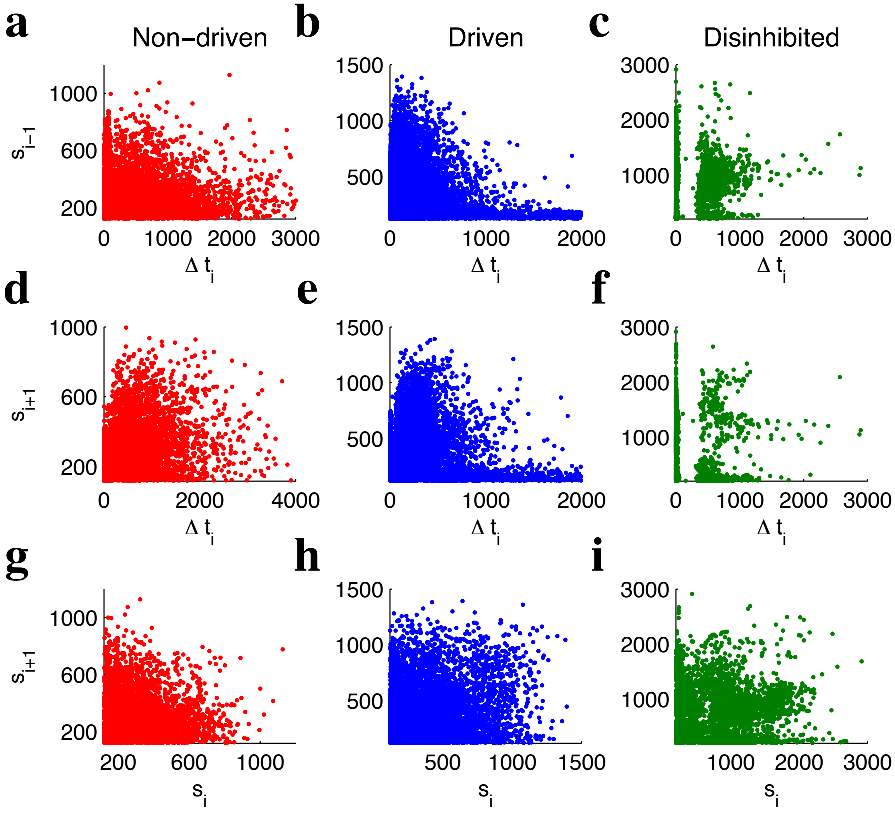


**Supplementary Fig. S2. Scatter plots.** Quiet times $\Delta t_i$ and sizes $s_{i-1}$ of preceding avalanches: (a) Non-driven sample; (b) Driven sample; (c) Disinhibited sample. Quiet times $\Delta t_i$ and sizes $s_{i+1}$ of following avalanches: (d) Non-driven sample; (e) Driven sample; (f) Disinhibited sample. Sizes of consecutive avalanches: (g) Non-driven sample; (h) Driven sample; (i) Disinhibited sample.

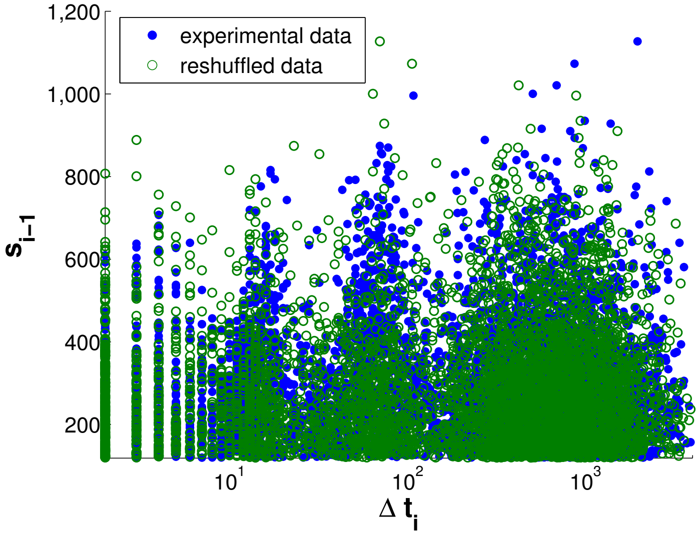


**Supplementary Fig. S3. Scatter plots of experimental data sets are comparable to the ones of uncorrelated data sets.** Quiet times $\Delta t_i$ and sizes $s_{i-1}$ of preceding avalanches in a non-driven sample. Similar behaviour is observed in all samples. Uncorrelated data set is obtained by reshuffling avalanche sizes while keeping fixed their starting and ending times.

# Correlations between the size $s_i$ and the following quiet time $\Delta t_i$

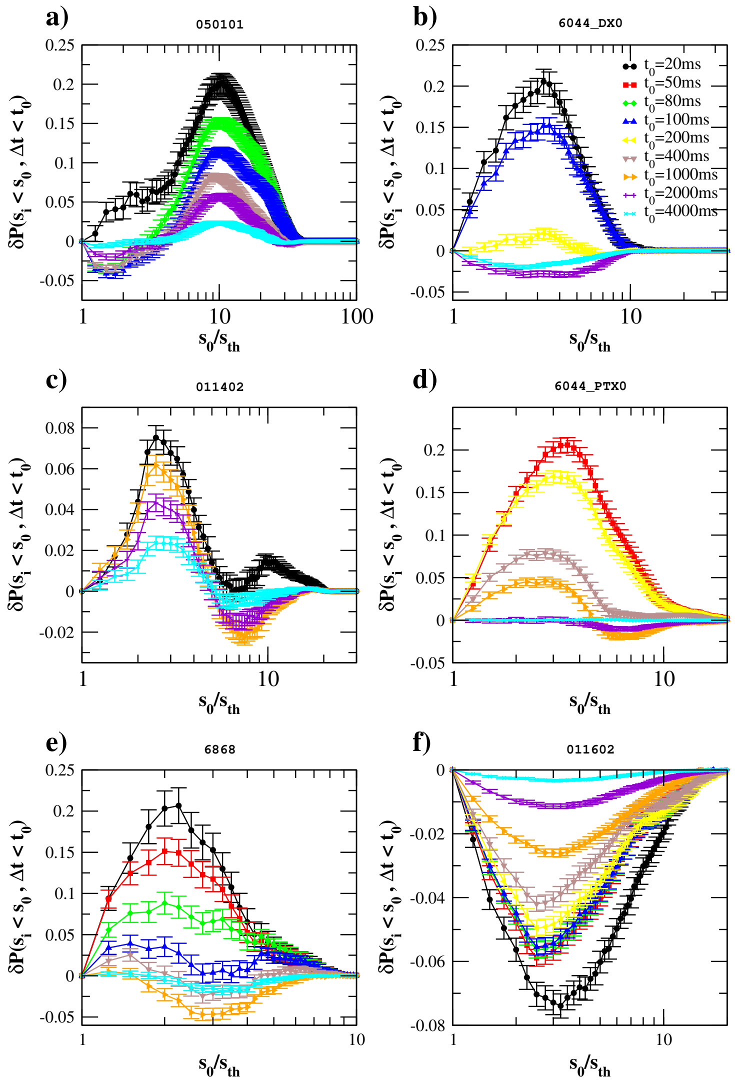


**Supplementary Fig. S4. Correlations between the size $s_i$ and the following quiet time $\Delta t_i$. Non-driven cultures.** The quantity $\delta P(s_i < s_0, \Delta t_i < t_0)$ for different non-driven samples. The error bar on each point $(s_0, t_0)$ is the standard deviation $\sigma(s_i < s_0, \Delta t_i < t_0)$. $s_{th} = 120\mu V$ in all samples except c) and f), for which $s_{th} = 250\mu V$. Samples a), b), c), d) and e) follow the average behavior discussed in Section 2.1, namely small avalanches tend to be followed by short quiet times. Deviations are observed in samples f), for which small avalanches are anti-correlated with short following quiet times (see Methods). Here we notice that, in contrast with other cultures, for this sample the quiet time distributions $P(\Delta t; s_c)$ do not show a $\theta$ peak, whereas they have a local maximum around 1000 ms (Fig. S13).

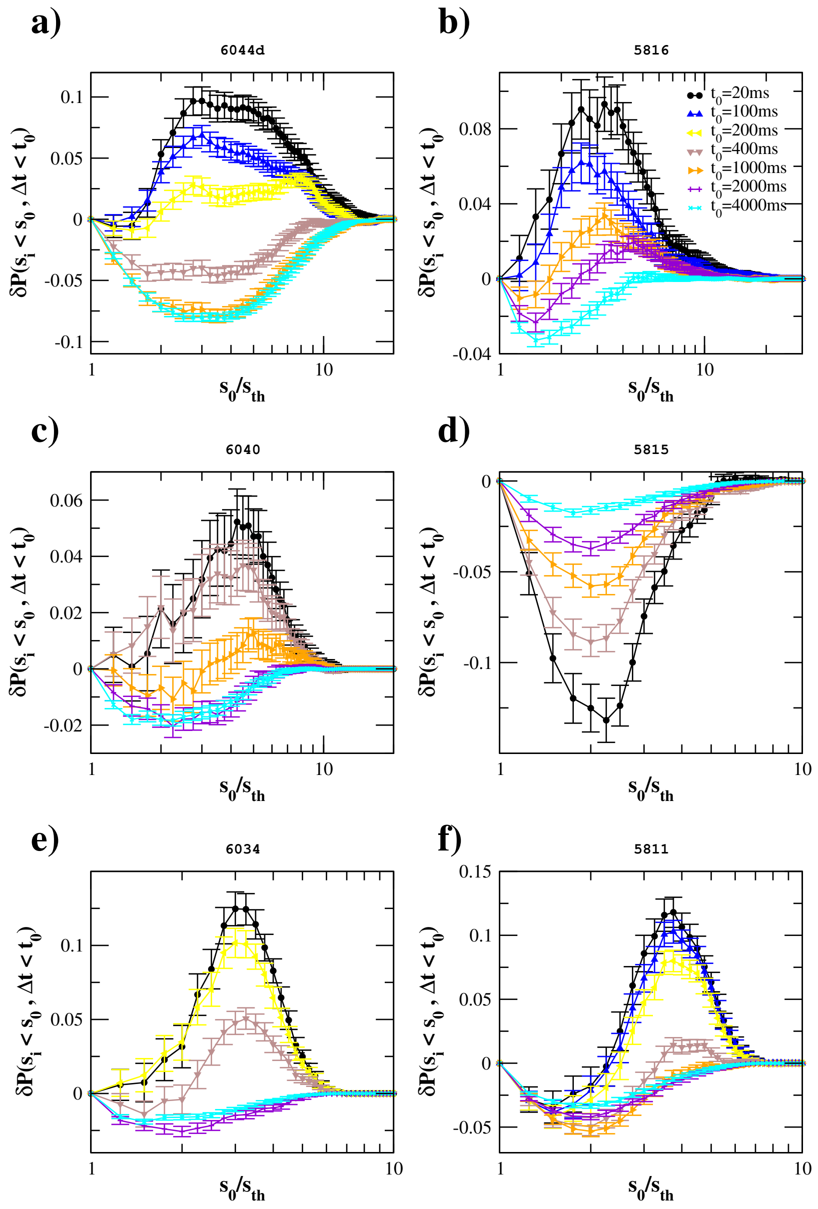


**Supplementary Fig. S5. Correlations between the size $s_i$ and the following quiet time $\Delta t_i$. Driven cultures.** The quantity $\delta P(s_i < s_0, \Delta t_i < t_0)$ for different driven samples. The error bar on each point $(s_0, t_0)$ is the standard deviation $\sigma(s_i < s_0, \Delta t_i < t_0)$. $s_{th} = 200\mu V$ in b), d) and f), $s_{th} = 250\mu V$ in a), $s_{th} = 50\mu V$ in c) and $s_{th} = 120\mu V$ in e). Samples a), b), c), e) and f) follow the average behavior discussed in Section 2.1, namely the longer the quiet time the larger the following avalanche. Deviations are observed in samples d), for which small avalanches are anti-correlated with short preceding quiet times (see Methods). We notice that for this sample the quiet time distributions $P(\Delta t; s_c)$ do not show a $\theta$ peak, whereas they exhibit a broad peak around 1000 ms (Fig. S14).

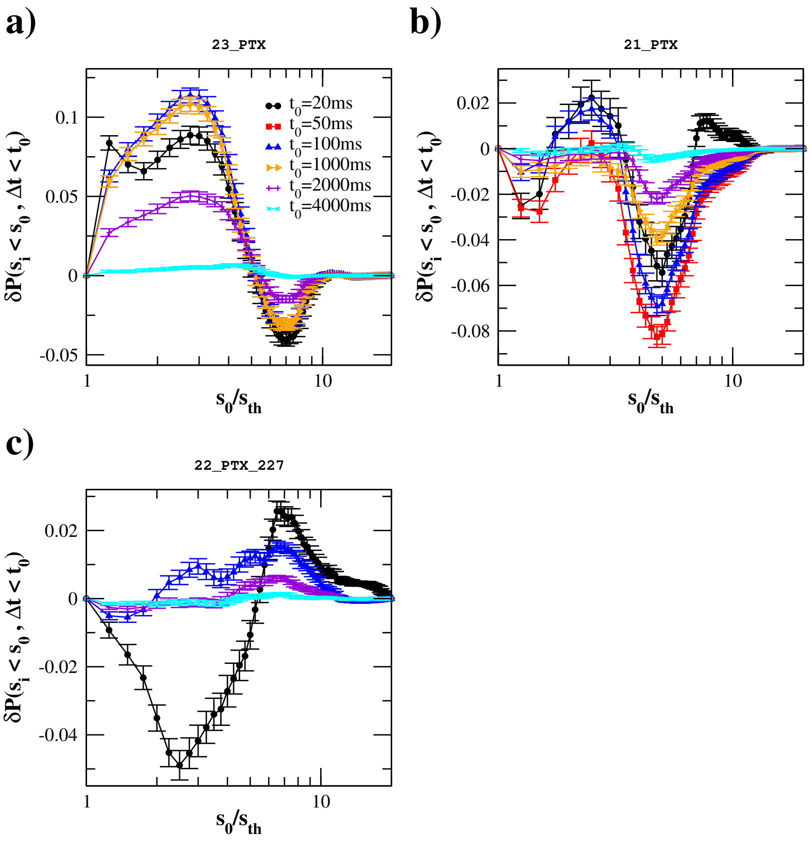


**Supplementary Fig. S6. Correlations between the size $s_i$ and the following quiet time $\Delta t_i$. Disinhibited cultures.** The quantity $\delta P(s_i < s_0, \Delta t_i < t_0)$ for different disinhibited (PTX) samples. The error bar on each point $(s_0, t_0)$ is the standard deviation $\sigma(s_i < s_0, \Delta t_i < t_0)$. $s_{th} = 200\mu V$ in a), c) and d) and $s_{th} = 120\mu V$ in b). The average behavior discussed in Section 2.1 arises from samples a) and b). Deviation are observed in c), whose quiet time distributions $P(\Delta t; s_c)$, in contrast with samples a) and b), do not exhibit a $\theta$ peak (Fig. S15).

# Correlations between the size $s_{i+1}$ and the preceding quiet time $\Delta t_i$

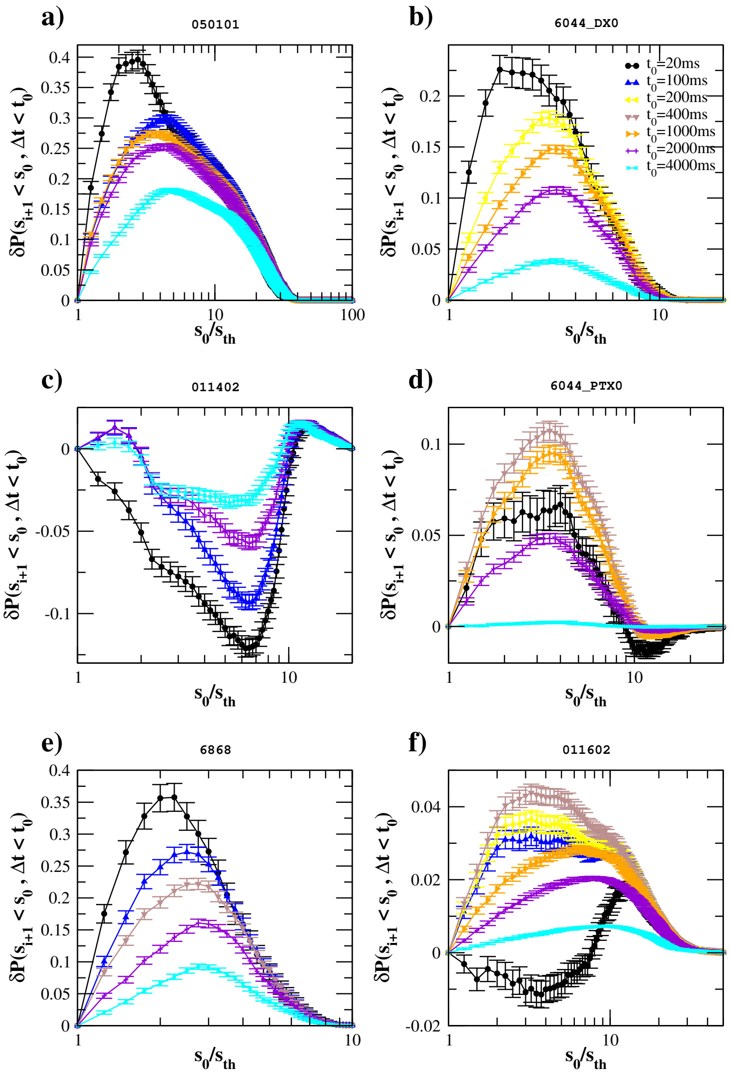


**Supplementary Fig. S7. Correlations between the size $s_{i+1}$ and the preceding quiet time $\Delta t_i$. Non-driven cultures.** The quantity $\delta P(s_{i+1} < s_0, \Delta t_i < t_0)$ for different non-driven samples. The error bar on each point $(s_0, t_0)$ is the standard deviation $\sigma(s_{i+1} < s_0, \Delta t_i < t_0)$. $s_{th} = 120\mu V$ in all samples except c) and f), for which $s_{th} = 250\mu V$. Samples a), b), d) and e) follow the average behavior described in Section 2.2. Sample f) slightly deviates from this behavior for short $\Delta t$s. Anti-correlation between short quiet times and small following avalanches is instead observed in panel c). Samples in c) and f) do not show a $\theta$ peak in the distributions $P(\Delta t; s_c)$ (Fig. S13).

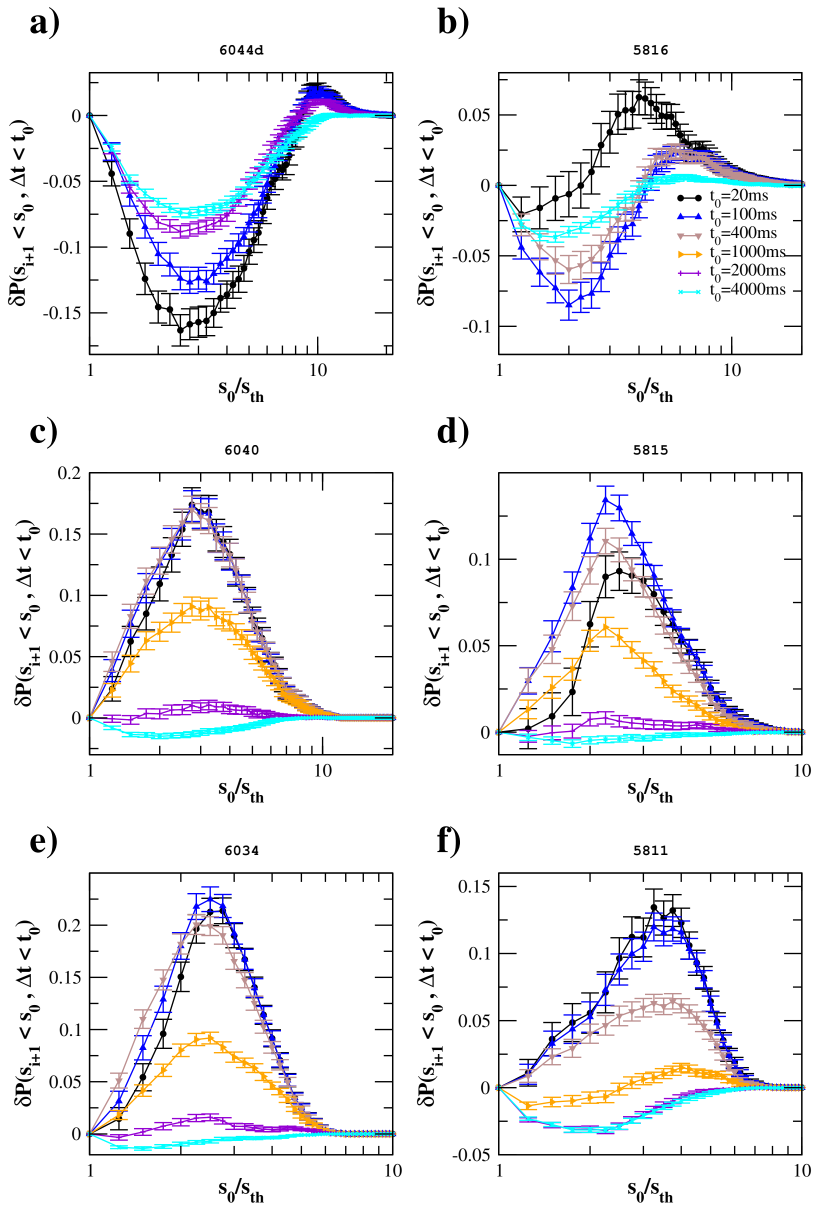


**Supplementary Fig. S8. Correlations between the size $s_{i+1}$ and the preceding quiet time $\Delta t_i$. Driven cultures.** The quantity $\delta P(s_{i+1} < s_0, \Delta t_i < t_0)$ for different driven samples. The error bar on each point $(s_0, t_0)$ is the standard deviation $\sigma(s_{i+1} < s_0, \Delta t_i < t_0)$. $s_{th} = 200\mu V$ in b), d) and f), $s_{th} = 250\mu V$ in a), $s_{th} = 50\mu V$ in c) and $s_{th} = 120\mu V$ in e). All samples follow the average behavior described in Section 2.2, except a) and b). In this case deviations from the average behavior cannot be related to the absence of a $\theta$ peak in the distributions $P(\Delta t; s_c)$ (Fig. S14).

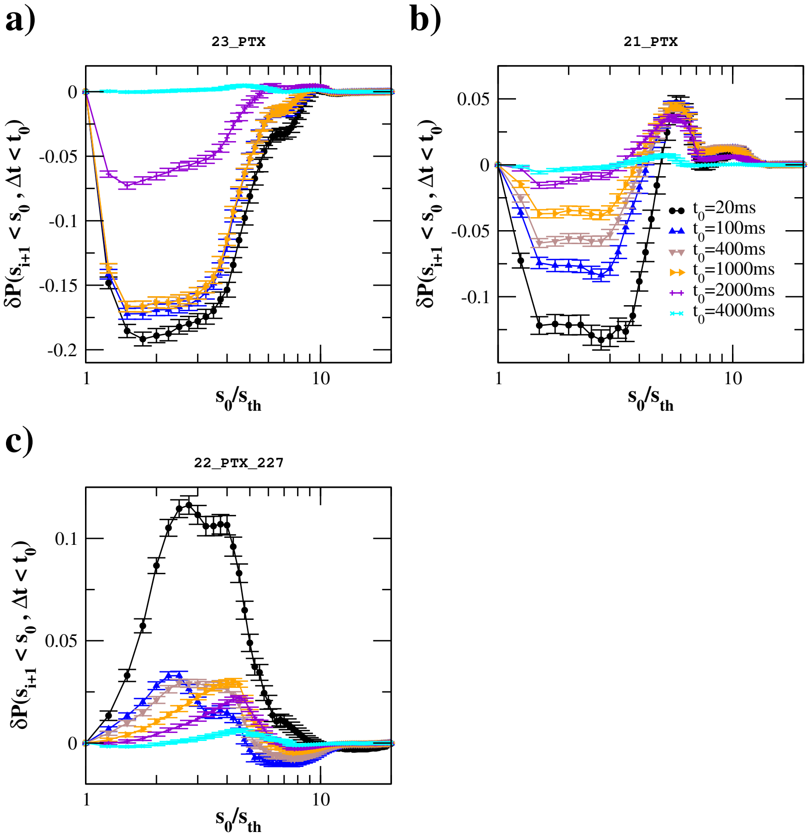


**Supplementary Fig. S9. Correlations between the size $s_{i+1}$ and the preceding quiet time $\Delta t_i$. Disinhibited cultures.** The quantity $\delta P(s_{i+1} < s_0, \Delta t_i < t_0)$ for different disinhibited (PTX) samples. The error bar on each point $(s_0, t_0)$ is the standard deviation $\sigma(s_{i+1} < s_0, \Delta t_i < t_0)$. $s_{th} = 200\mu V$ in a), c) and d) and $s_{th} = 120\mu V$ in b). Disinhibited cultures. Samples a) and b) follow the average behavior described in Section 2.2. Conversely sample c) follows the same behavior found in the normal condition (see Fig. S7).

# Correlations between consecutive avalanche sizes

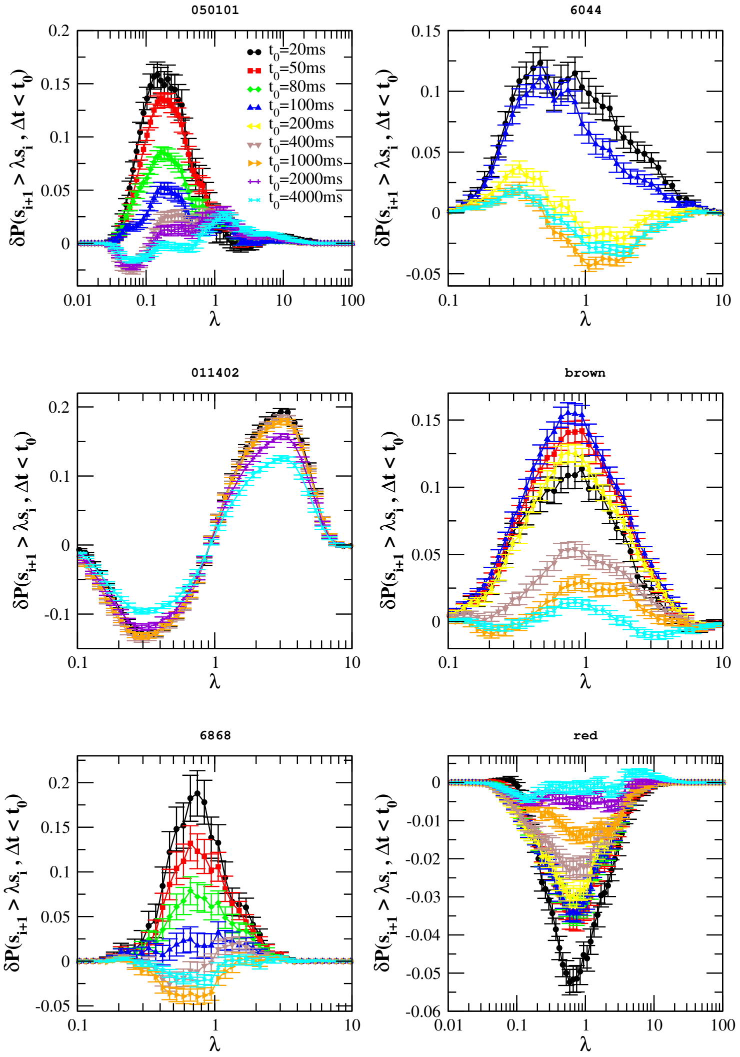


**Supplementary Fig. S10. Correlations between consecutive avalanche sizes. Non-driven cultures.** The quantity $\delta P(s_{i+1} > \lambda s_i, \Delta t_i < t_0)$ as a function of $\lambda$ for different values of $t_0$ and different non-driven samples. The error bar on each data point is the standard deviation $\sigma(s_{i+1} > \lambda s_i, \Delta t_i < t_0)$. $s_{th} = 120\mu V$ in all samples except c) and f), for which $s_{th} = 250\mu V$. Samples a), b), d) and e) follow the average behavior described in Section 2.3. Samples c) and f), which do not show a $\theta$ peak in the distributions $P(\Delta t; s_c)$ (Fig. S13), significantly deviate from the average behavior. In particular in c) an avalanche tends to be larger than the previous one, independently of $\Delta t$.

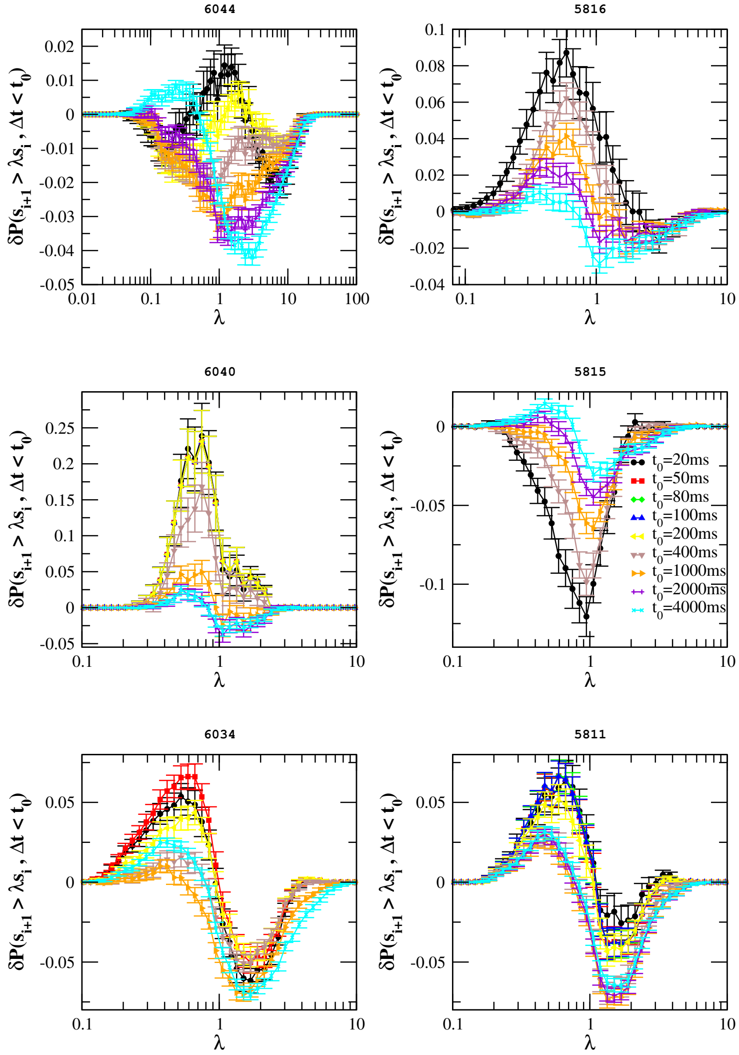


**Supplementary Fig. S11. Correlations between consecutive avalanche sizes. Driven cultures.** The quantity $\delta P(s_{i+1} > \lambda s_i, \Delta t_i < t_0)$ as a function of $\lambda$ for different values of $t_0$ and different driven samples. The error bar on each point $(s_0, t_0)$ is the standard deviation $\sigma(s_{i+1} > \lambda s_i, \Delta t_i < t_0)$. $s_{th} = 200\mu V$ in b), d) and f), $s_{th} = 250\mu V$ in a), $s_{th} = 50\mu V$ in c) and $s_{th} = 120\mu V$ in e). Deviations from the average behavior discussed in Section 2.3 are found in a), c) and d) and can be related to the absence of a $\theta$ peak in the distributions $P(\Delta t; s_c)$ (Fig. S14). In particular in a) and c) the maximum for $t_0 = 20$ ms is located at $\lambda > 0$, meaning that, for close in time avalanches, the following avalanche tends to be larger than the previous one.

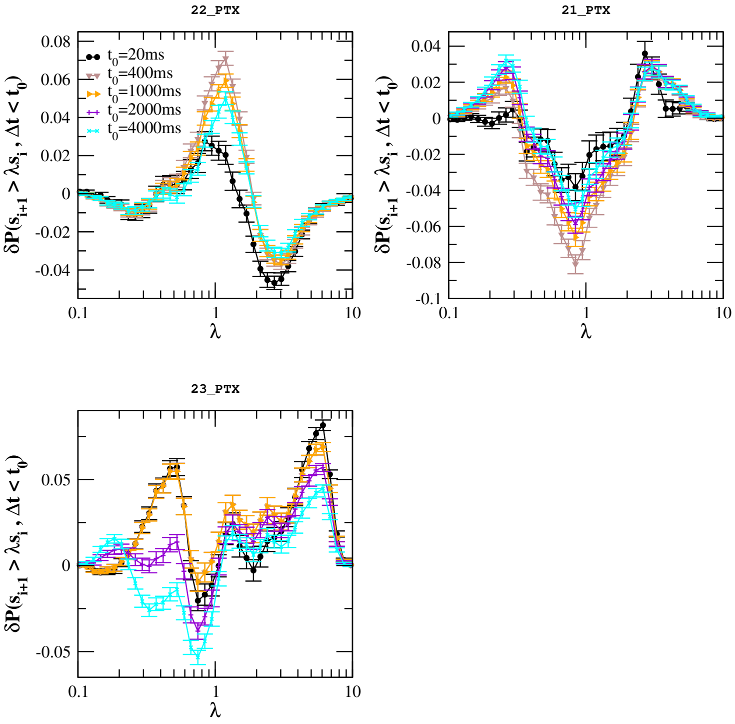


**Supplementary Fig. S12. Correlations between consecutive avalanche sizes. Disinhibited cultures.** The quantity $\delta P(s_{i+1} > \lambda s_i, \Delta t_i < t_0)$ as a function of $\lambda$ for different values of $t_0$ and different disinhibited (PTX) samples. The error bar on each point $(s_0, t_0)$ is the standard deviation $\sigma(s_{i+1} > \lambda s_i, \Delta t_i < t_0)$. $s_{th} = 200\mu V$ in a), c) and d) and $s_{th} = 120\mu V$ in b). Disinhibited cultures. In all samples $\delta P(s_{i+1} > \lambda s_i, \Delta t_i < t_0)$ has a maximum for $\lambda > 0$, also for small $t_0$ values. This implies that, independently of the time separation between consecutive avalanches, there is a significant probability that the following avalanche is lager than the previous one.

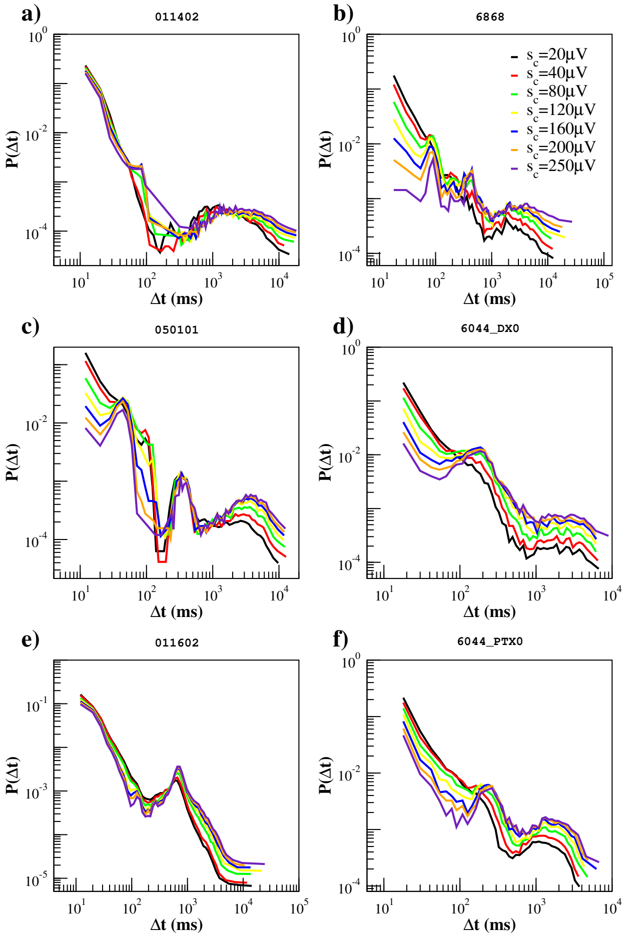


**Supplementary Fig. S13. Distributions $P(\Delta t, s_c)$ of quiet times between consecutive avalanches of size larger than a given threshold $s_c$ (see *Lombardi et al.* (2014), Front. Syst. Neurosci. 8:204) for different non-driven cultures.** Already for $s_c = 80\,\mu\text{V}$, distributions clearly exhibit one or more additional peaks. Beside the one at large time scales, $\Delta t \simeq 1000 - 2000$ ms, which is related with the characteristic time of up-state recurrence, at least one peak between 60 ms and 250 ms is always visible. The $\theta$ peak ($\sim$ 100-300 ms) is particularly pronounced in b), c), d) and f), less in a) and e).

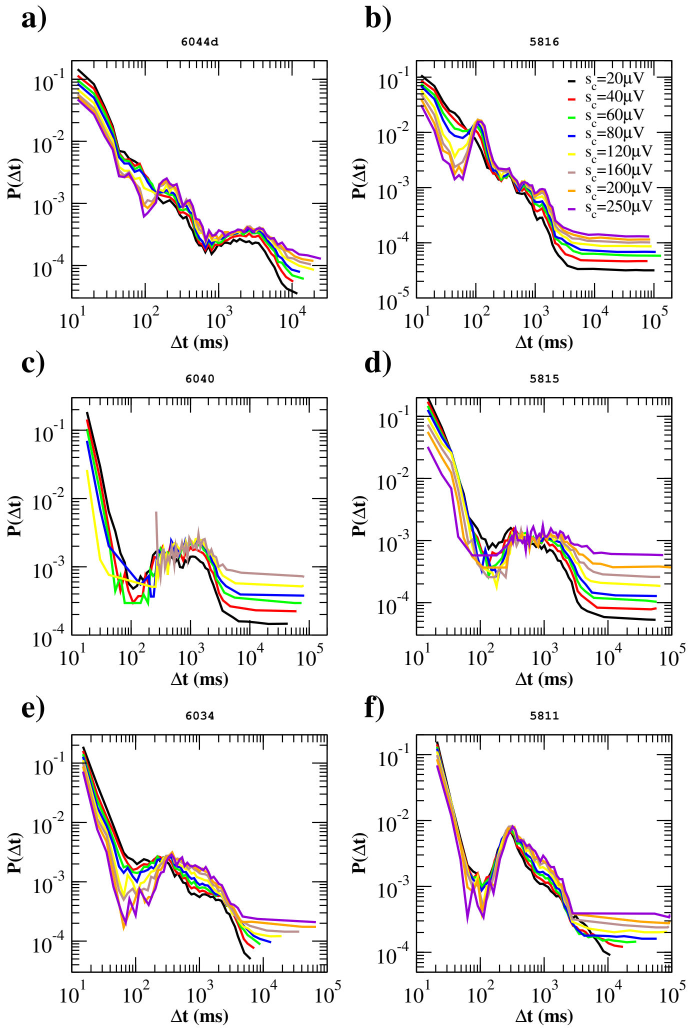


**Supplementary Fig. S14. Distributions $P(\Delta t, s_c)$ of quiet times between consecutive avalanches of size larger than a given threshold $s_c$ for different driven cultures.** A $\theta$ peak (100-300 ms) is clearly visible in a), b), e) and f). Panel c) and d) shows a broad peak around 1000 ms, related to slow oscillations.

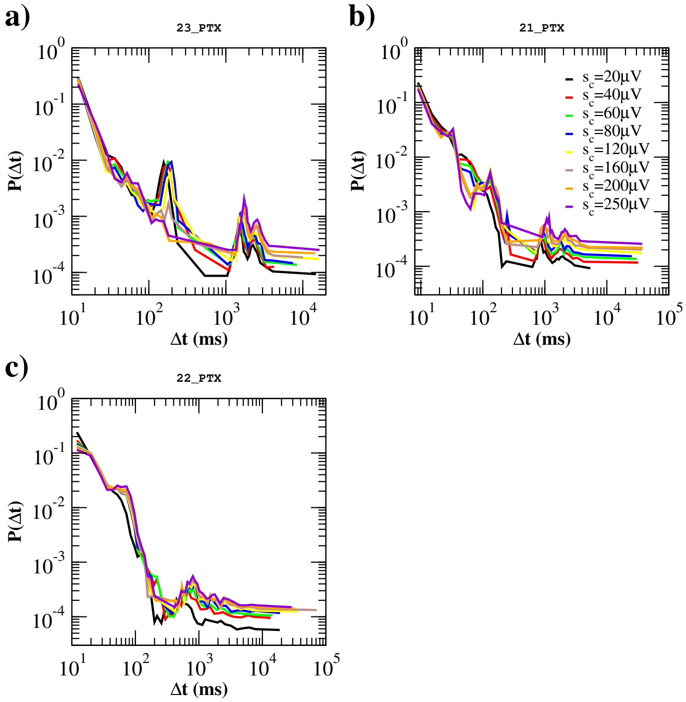


**Supplementary Fig. S15. Distributions $P(\Delta t, s_c)$ of quiet times between consecutive avalanches of size larger than a given threshold $s_c$ for different disinhibited (PTX) cultures.** Panel a) and b) exhibit a $\theta$ (100-300 ms) peak, which emerges already for small $s_c$ values. On the other hand, sample in panel c) does not display a pronounced peak that can be related to $\theta$ oscillations.
